# Cleanliness and Health

**Published:** 1891-08

**Authors:** 


### CLEANLINESS AND HEALTH.

If asked, said Dr. Alfred Hill to the members of the British Medical Association, lately, what have been the principal agencies by which the past triumphs of preventive medicine have been achieved, there would be little hesitation in answering that the majority of the scourges which have afflicted mankind and been overcome have yielded to cleanliness. The plague and the mediæval epidemics were banished by cleanliness. Typhus, thanks to the labors of Howard, was influenced by cleanliness as if by magic. Cholera, so fatal in its first visitations, before its favoring conditions were understood, wrought terrible havoc where filth conditions prevailed. Two hundred years ago the annual death rate of England was eighty per 1000 of the population ; now it is but twenty.

